# Supplementary material for: Modulation of tooth regeneration through opposing responses to Wnt and BMP signals in teleosts
Source: Development. 2023 Dec 7;150(23):dev202168. doi: 10.1242/dev.202168 (PMC10730089; doi:10.1242/dev.202168)
Supplement: Supplementary information [file develop-150-202168-s1.pdf]

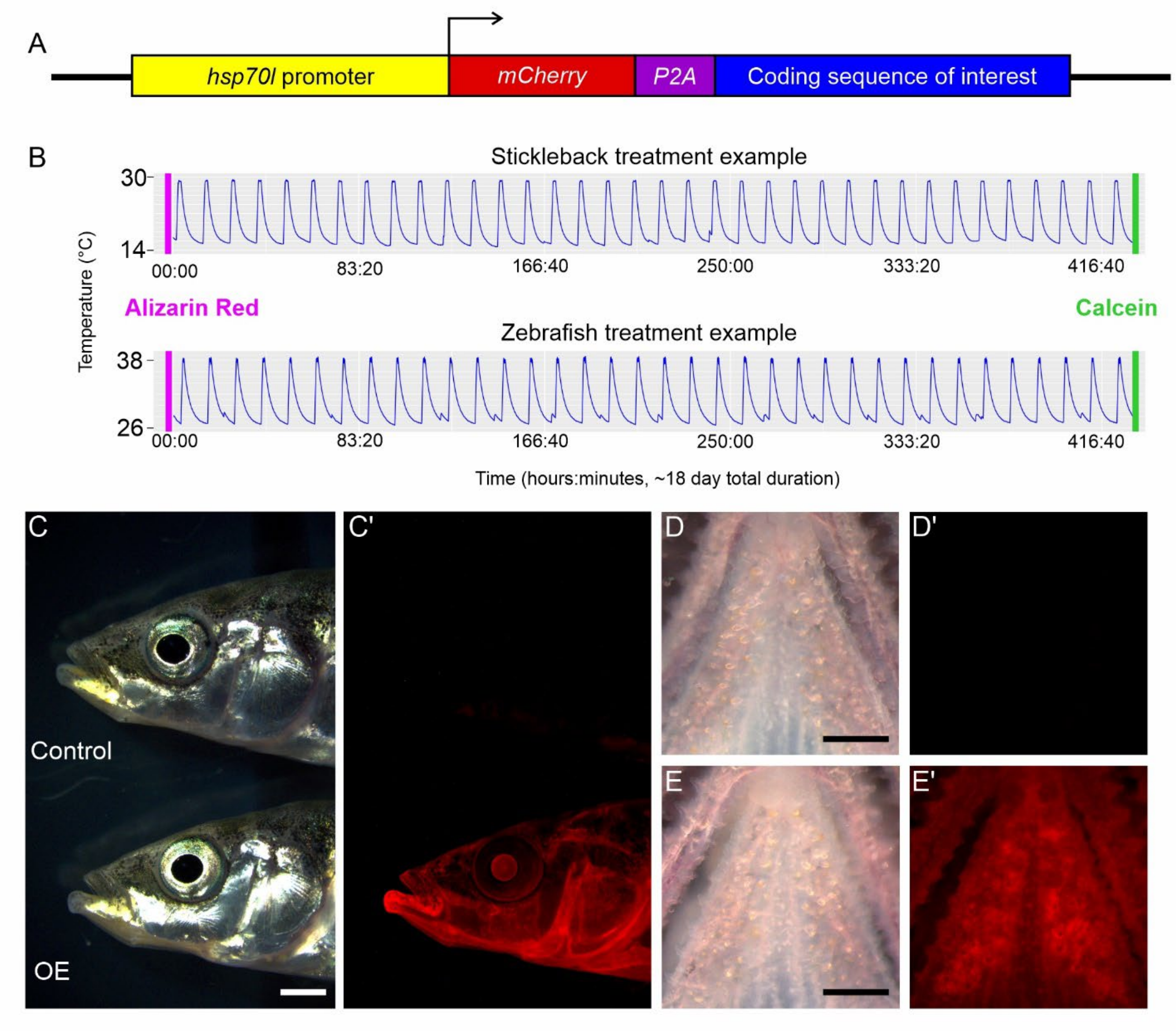

Fig. S1. Heat shock method overview. A. Schematic of OE transgenes. The zebrafish *hsp70l* promoter drives an mCherry-P2A-CDS transgene, where "CDS" is one of seven full-length coding sequences of interest (see Methods and Table S2). P2A is a 22 amino acid sequence that cleaves between residues 20 and 21, separating the mCherry fluorophore from the translated CDS. **B.** Example temperature profiles for heat shock treatments in sticklebacks (top) and zebrafish (bottom). Alizarin pulse and calcein chase are shown as pink and green bars, respectively. **C.** Left lateral images of sticklebacks 24 hours after a single heat shock. Control (sibling without transgene) on top, overexpression transgene positive (OE) fish on bottom (*Bmp6* OE in this example). C' shows red channel fluorescence, allowing visualization of the transgene in the OE treatment. **D and E.** Dissecting the ventral tooth plate (VTP) fish revealed mCherry present throughout the tooth field in the OE treatment condition (anterior to top). B' and C' show red channel fluorescence of B and C. Scale bar in A: 2 mm, B and C: 500 µm.

Development • Supplementary information

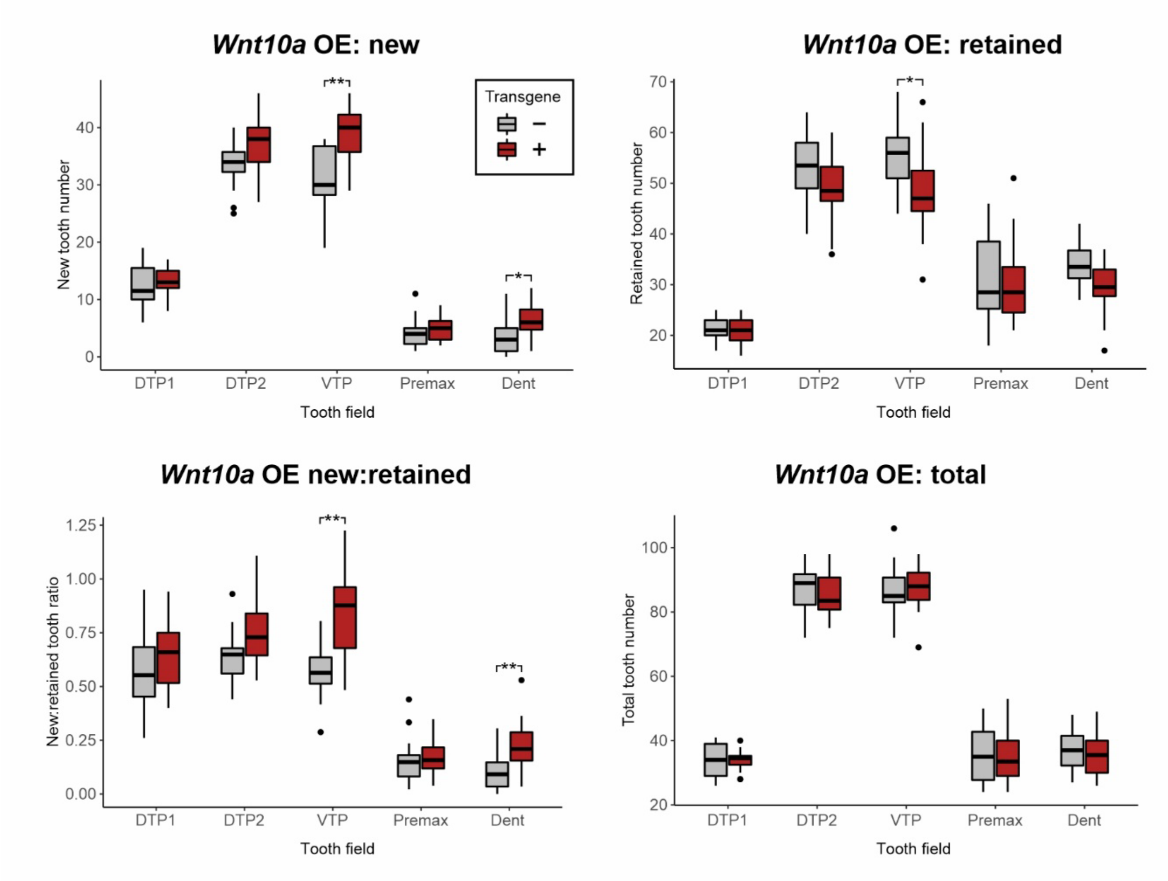

Fig. S2. Results of stickleback *Wnt10a* OE parsed by tooth field reveals strongest effects on ventral tooth fields (ventral tooth plate and dentary). Stickleback pharyngeal teeth are housed in three discrete fields: the 1st dorsal tooth plate 1 (DTP1), the 2nd dorsal tooth plate (DTP2), and the ventral tooth plate (VTP). In the oral jaws, sticklebacks have teeth on their premaxilla (Premax) and dentary (Dent) bones. Each group of graphs shows new, retained, the new:retained ratio, and total tooth number broken down by tooth field type (the sum of left and right halves per fish). **P*<0.05, ***P*<0.01, ****P*<0.001 (Wilcoxon Rank-Sum tests, Benjamini-Hochberg corrected *P* for multiple hypothesis testing). Insignificant results (*P*>0.05) are unannotated. Boxes represent the 25th-75th percentiles, and the median is shown as a gray bar.

Development • Supplementary information

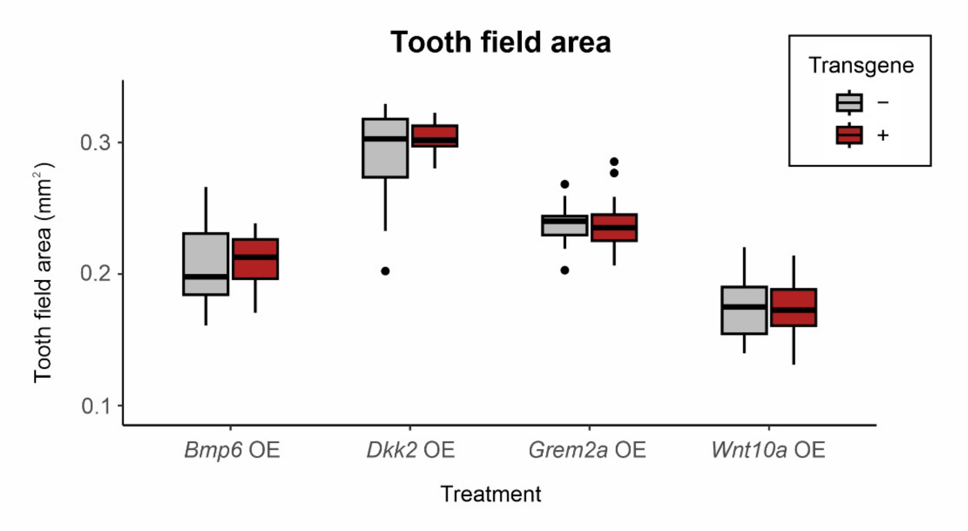

Fig. S3. VTP area did not demonstrate significant changes under any OE experiment. Box and whisker plots showing left VTP area (mm^2) for all sticklebacks that underwent a heat shock treatment. Area values were corrected for standard length. No significant differences were observed in any experiment (all P>0.4, Wilcoxon Rank-Sum tests). Boxes represent the 25th-75th percentiles, and the median is shown as a gray bar.

Development • Supplementary information

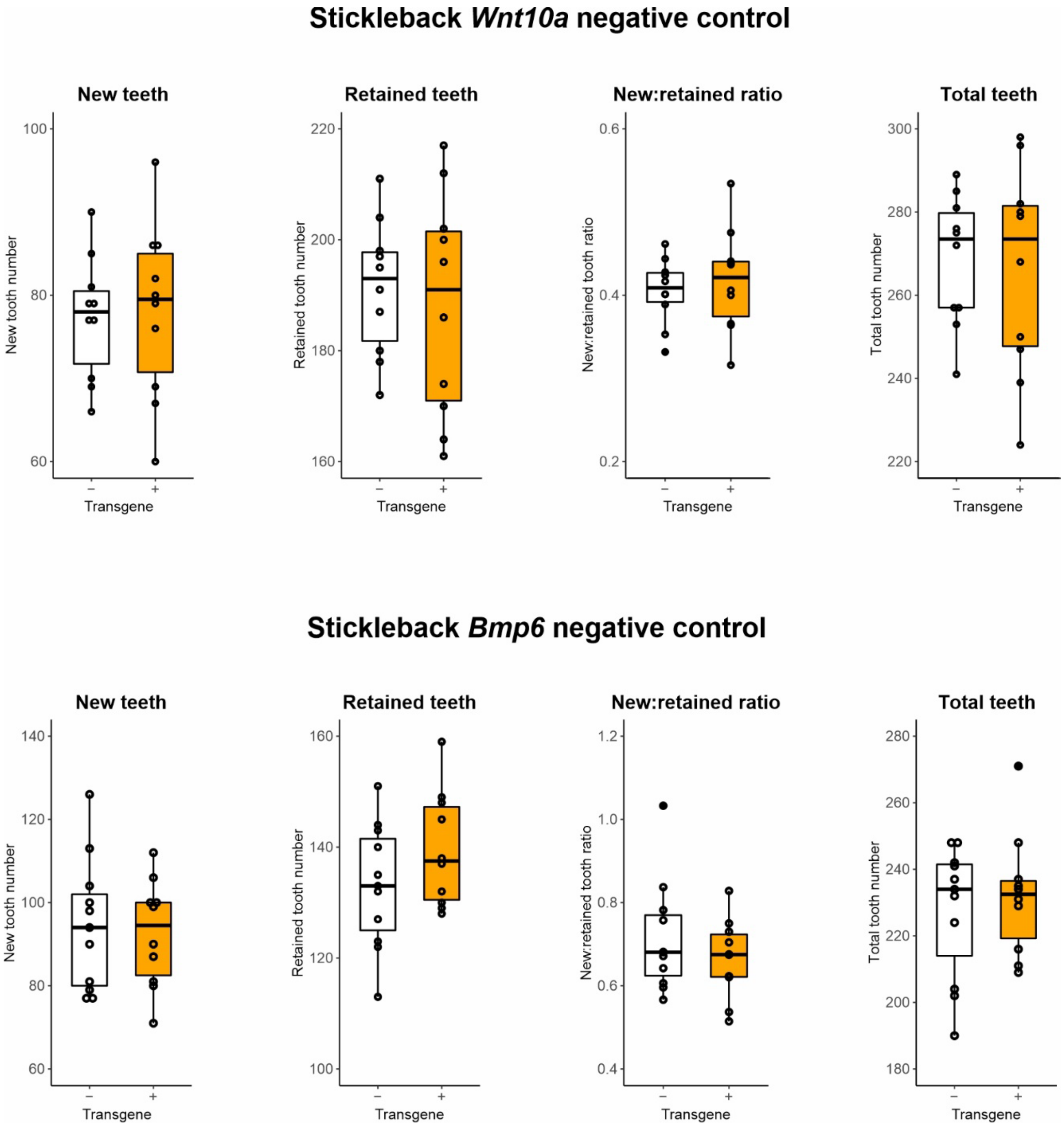

Fig. S4. Negative control experiments suggest inducible transgenes in the absence of heat shocks have little to no effect on tooth replacement dynamics. The same pulse-chase interval (18 days, see Methods) with no intervening heat shocks was applied to full siblings of the fish in the experiments summarized in Fig. 3A and Fig. 6A, testing for whether simply carrying the transgene in the absence of any heat shock is associated with changes in the number of new teeth, retained teeth, the new:retained ratio, or total teeth; no such deviations were detected in this negative control experiment for any of these four variables assessed (all $P>0.90$ and $P>0.26$ for *Wnt10a* and *Bmp6* negative control experiments, respectively, Wilcoxon Rank-Sum tests, Benjamini-Hochberg adjusted for multiple hypothesis testing. No “stalled” tooth germs were observed in either negative control assay. Boxes represent the 25th-75th percentiles, and the median is shown as a gray bar.

Development • Supplementary information

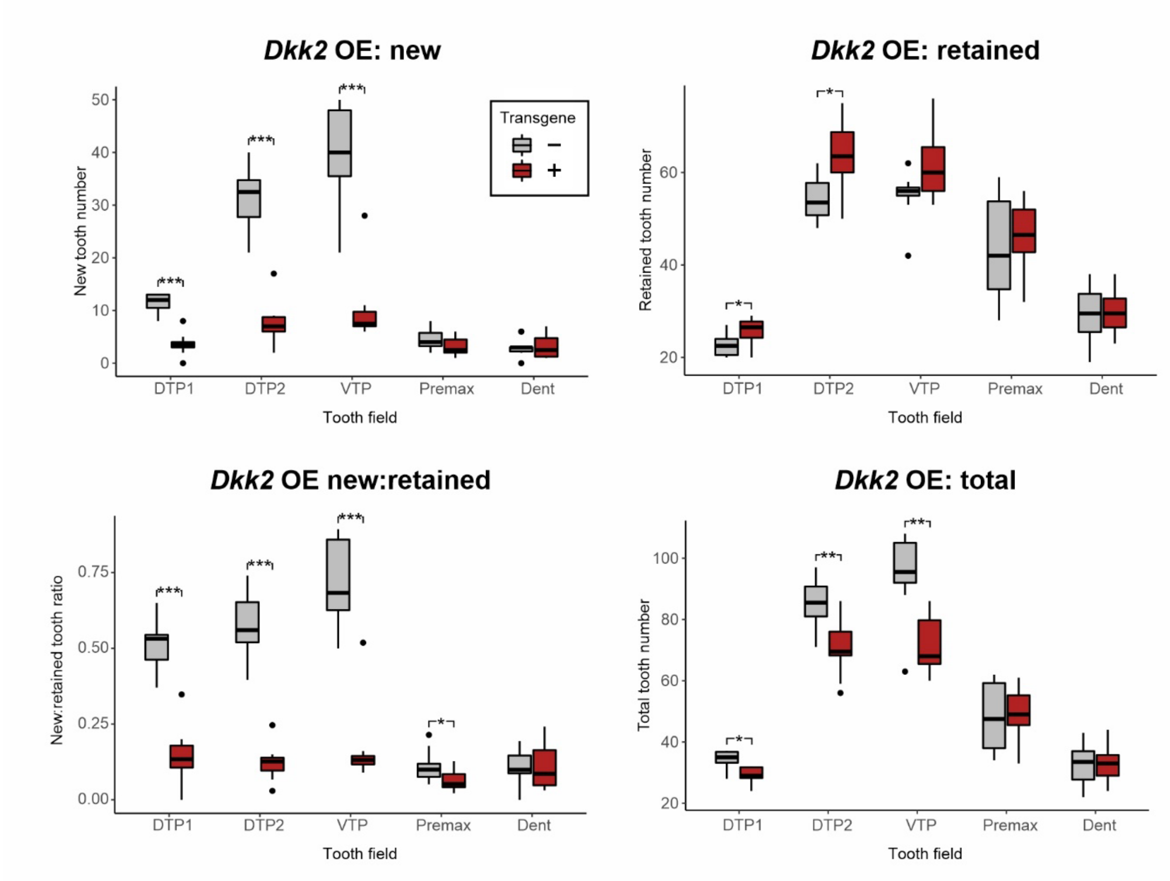

Fig. S5. Results of stickleback *Dkk2* OE parsed by tooth field reveals effects primarily on pharyngeal teeth. Stickleback pharyngeal teeth are housed in three discrete fields: the 1st dorsal tooth plate 1 (DTP1), the 2nd dorsal tooth plate (DTP2), and the ventral tooth plate (VTP). In the oral jaws, sticklebacks have teeth on their premaxilla (Premax) and dentary (Dent) bones. Each group of graphs shows new, retained, the new:retained ratio, and total tooth number broken down by tooth field type (the sum of left and right halves per fish). **P*<0.05, ***P*<0.01, ****P*<0.001 (Wilcoxon Rank-Sum tests, Benjamini-Hochberg corrected *P* for multiple hypothesis testing). Insignificant results (*P*>0.05) are unannotated. Boxes represent the 25th-75th percentiles, and the median is shown as a gray bar.

Development • Supplementary information

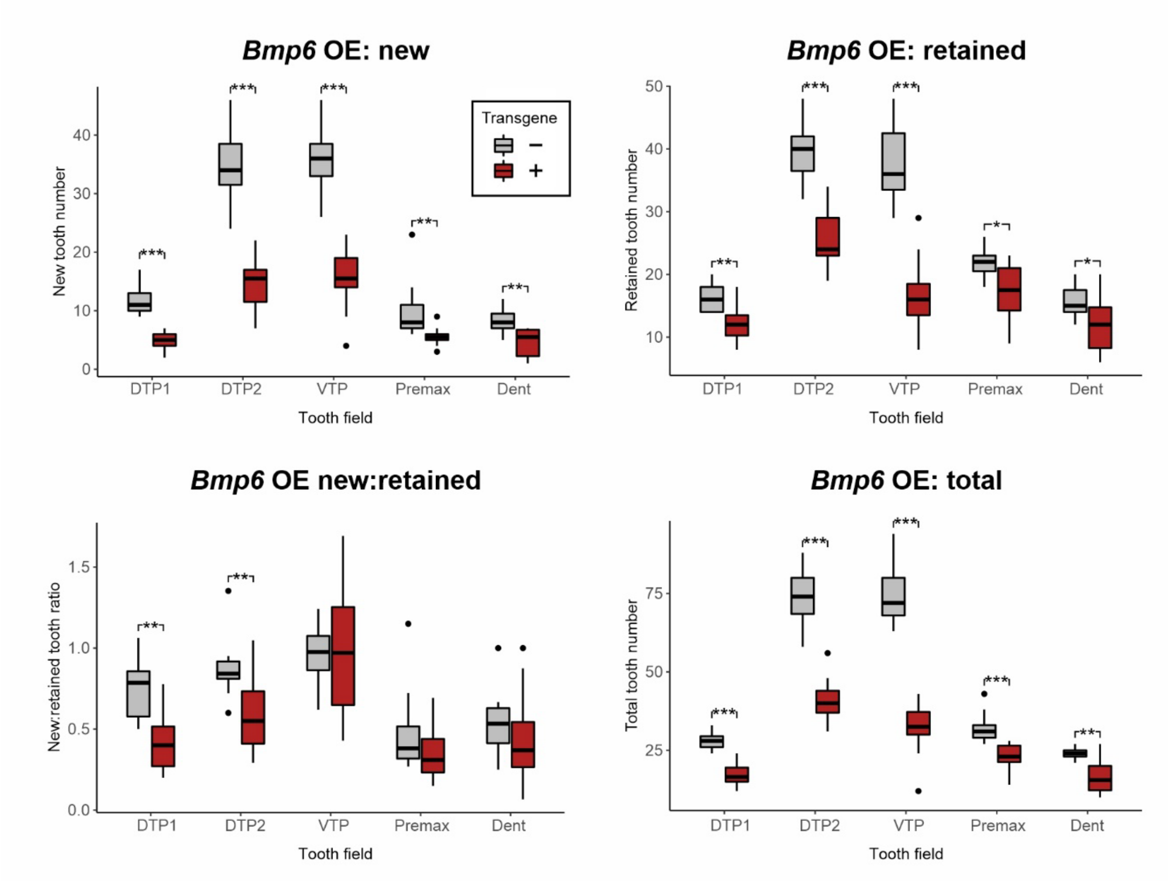

Fig. S6. Results of stickleback *Bmp6* OE parsed by tooth field reveals more pronounced effects on pharyngeal teeth than oral teeth. Stickleback pharyngeal teeth are housed in three discrete fields: the 1st dorsal tooth plate 1 (DTP1), the 2nd dorsal tooth plate (DTP2), and the ventral tooth plate (VTP). In the oral jaws, sticklebacks have teeth on their premaxilla (Premax) and dentary (Dent) bones. Each group of graphs shows new, retained, the new:retained ratio, and total tooth number broken down by tooth field type (the sum of left and right halves per fish). *P<0.05, **P<0.01, ***P<0.001 (Wilcoxon Rank-Sum tests, Benjamini-Hochberg corrected P for multiple hypothesis testing). Insignificant results (P> 0.05) are unannotated. Boxes represent the 25th-75th percentiles, and the median is shown as a gray bar.

Development • Supplementary information

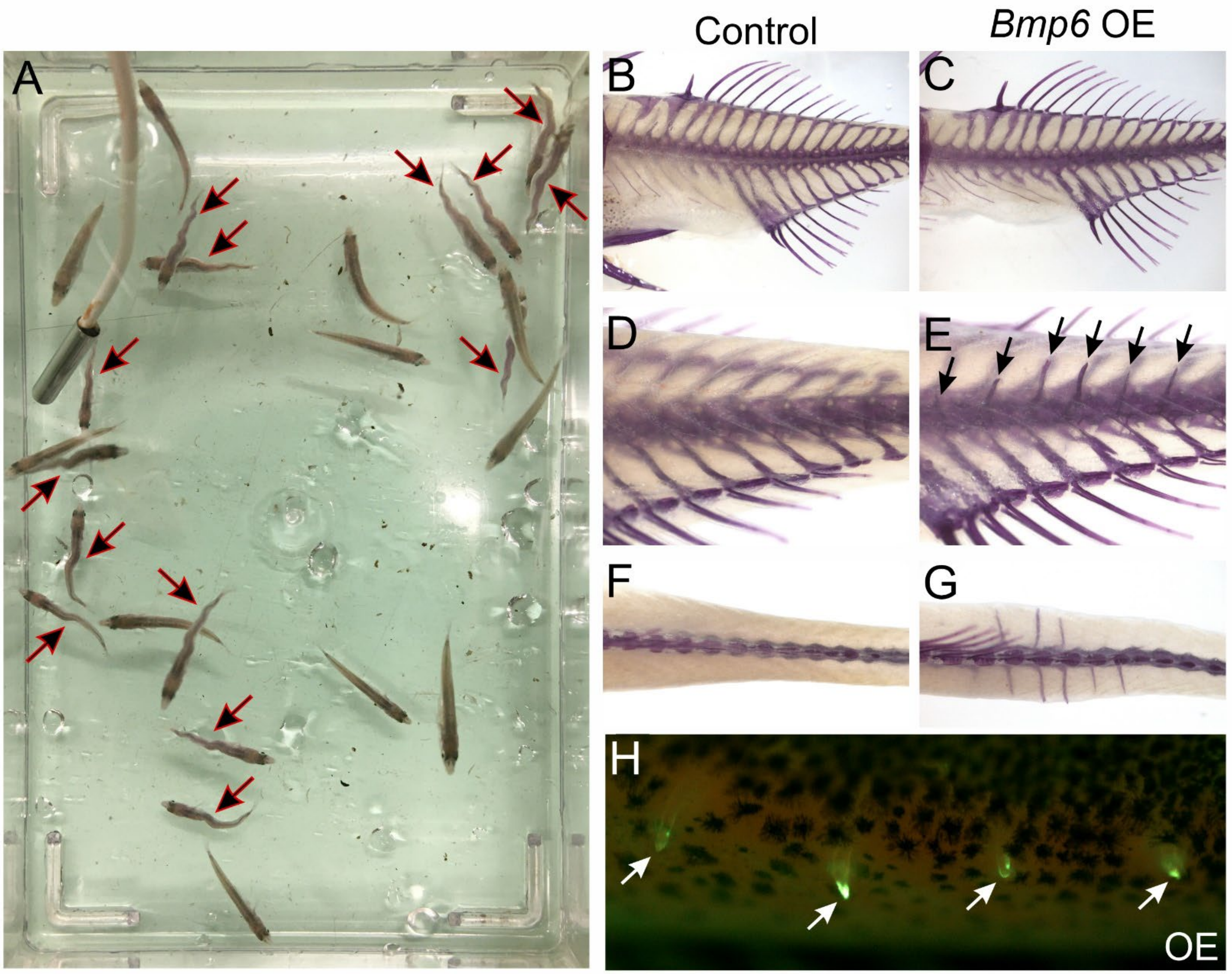

Fig. S7. Axial bending and ectopic rib-like bony protrusions arising from *Bmp6* OE. 14/14 OE fish had bends along their primary axis (arrows in A), 8/14 had some number of bony body spikes (black arrows in C, white arrows in D), all of which were confined to caudal vertebrae 3-8. B-G show fish skeletons that were re-stained with Alizarin Red after the pulse-chase experiment to better visualize the skeleton. B and C show straight lateral view, D and E show oblique lateral views tilted ~30 degrees on the coronal axis, F and G show dorsal views. H shows a lateral image of a treatment animal immediately after the pulse-chase experiment, before re-staining, revealing that the new bone growth is indeed strongly marked by calcein, confirming that these body spikes arose during the OE treatment interval.

Development • Supplementary information

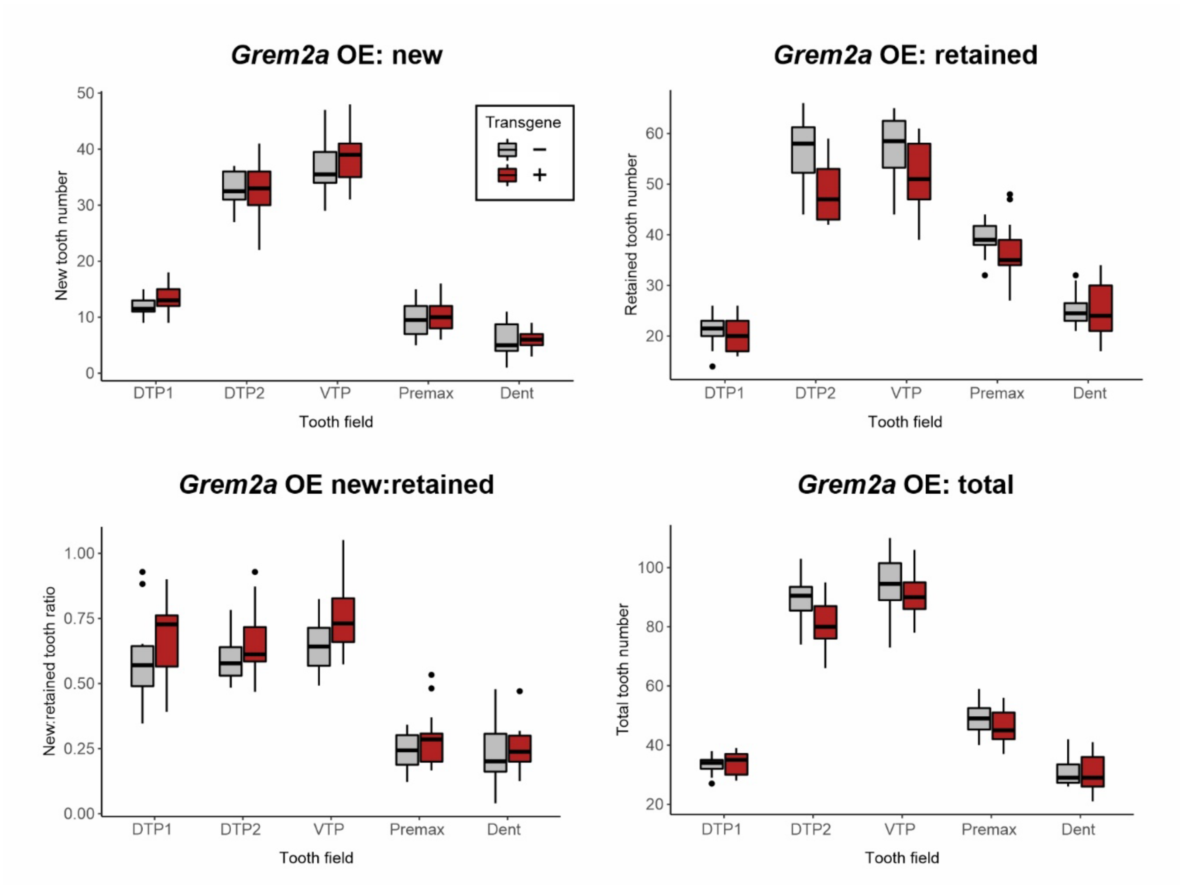

Fig. S8. Results of stickleback *Grem2a* OE parsed by tooth field reveals consistent but subtle effects on new, retained, and the new:retained ratio. Stickleback pharyngeal teeth are housed in three discrete fields: the 1st dorsal tooth plate 1 (DTP1), the 2nd dorsal tooth plate (DTP2), and the ventral tooth plate (VTP). In the oral jaws, sticklebacks have teeth on their premaxilla (Premax) and dentary (Dent) bones. Each group of graphs shows new, retained, the new:retained ratio, and total tooth number broken down by tooth field type (the sum of left and right halves per fish). All *P*>0.1279 (Wilcoxon Rank-Sum tests, Benjamini-Hochberg corrected *P* for multiple hypothesis testing). Boxes represent the 25th-75th percentiles, and the median is shown as a gray bar.

Development • Supplementary information

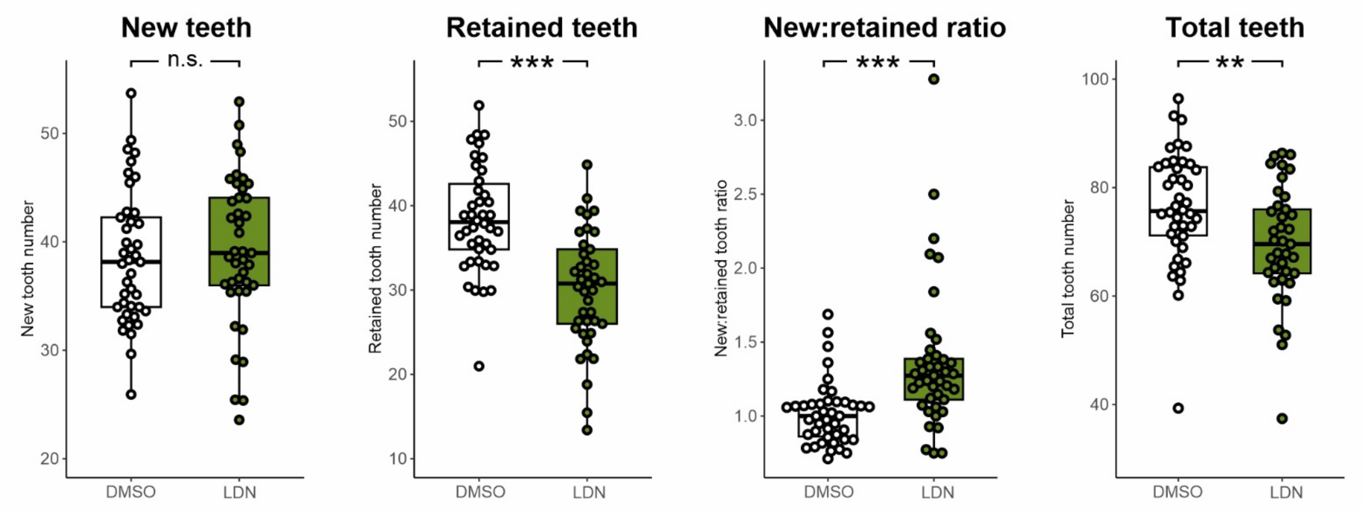

Fig. S9. **Effects of the BMP signaling inhibitor LDN-193189 on ventral tooth plates (VTP).** Using the same pulse-chase bone-labeling approach as in the OE experiments, we inhibited BMP signaling via LDN-193189 (n=42 control and 41 treatment fish). New tooth number did not significantly change (Wilcoxon Rank-Sum Benjamini-Hochberg adjusted *P*=0.34), retained teeth significantly decreased (*P*=4.9e-6), the new:retained tooth ratio significantly increased (*P*=1.5e-5), and total number of teeth decreased (*P*=0.0055). Boxes represent the 25th-75th percentiles, and the median is shown as a gray bar.

Development • Supplementary information

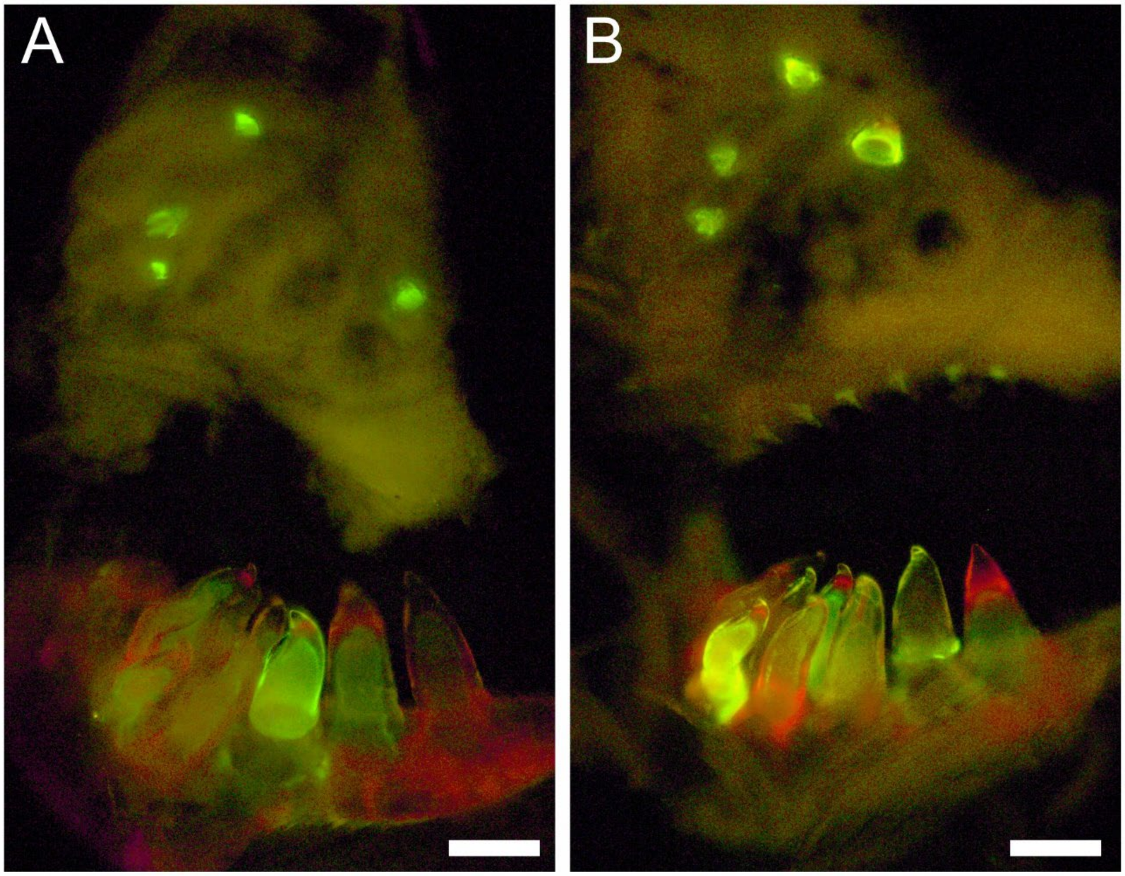

Fig. S10. Removal of epithelium with embedded tooth germs during zebrafish tooth scoring. Example images from the zebrafish *wnt10a* OE experiment are shown to illustrate this necessary step during the dissection process to obtain accurate scores in this species. Each image shows the left VTP of a zebrafish from the control (A) or *wnt10a* OE (B) condition. In order to score every tooth accurately, it is necessary to peel the epithelium (top of each image) from the underlying teeth and bone (bottom of each image). The small tooth germs typically remain embedded within the epithelium (green features at the top of each image). Thereafter, teeth are broken off from the tooth field, if necessary, especially in the dorsal and mediodorsal rows. See Methods. Scale bars 100 μm.

Table S1. Differential expression analysis of *Bmp6* OE reveals marked upregulation of Wnt and BMP inhibitors. The first sheet shows selected genes of interest specifically mentioned in the manuscript (namely Wnt and BMP inhibitors, BMP effectors, and Wnt ligands). The second sheet shows the results of all assessed genes. The log2-adjusted fold change column is color coded (negative numbers are blue and indicate higher expression in the OE condition, positive numbers are magenta and indicate higher expression in the control condition).

Available for download at
https://journals.biologists.com/dev/article-lookup/doi/10.1242/dev.202168#supplementary-data

Table S2. Coding sequences used in overexpression transgenes. Full nucleotide strings are provided for each of the overexpressed transgenes alongside their nearest GenBank accession number.

Available for download at
https://journals.biologists.com/dev/article-lookup/doi/10.1242/dev.202168#supplementary-data

Table S3. Stickleback tooth counts from OE experiments. Raw data arising from stickleback pulse-chase experiments formatted for analysis in R. **Stickleback tooth counts from LDN-193189 treatment.** Raw data arising from stickleback LDN-193189 treatment formatted for analysis in R.

Available for download at
https://journals.biologists.com/dev/article-lookup/doi/10.1242/dev.202168#supplementary-data

Table S4. Stickleback tooth counts from LDN-193189 treatment. Raw data arising from stickleback LDN-193189 treatment formatted for analysis in R.

Available for download at
https://journals.biologists.com/dev/article-lookup/doi/10.1242/dev.202168#supplementary-data

Table S5. Zebrafish tooth counts from OE experiments. Raw data arising from zebrafish pulse-chase experiments formatted for analysis in R.

Available for download at
https://journals.biologists.com/dev/article-lookup/doi/10.1242/dev.202168#supplementary-data

Table S6. *In situ* probe sequences. Full nucleotide strings are provided for each of the *in situ* hybridization probes alongside their nearest GenBank accession number. Probe templates are shown in the sense orientation.

Available for download at
https://journals.biologists.com/dev/article-lookup/doi/10.1242/dev.202168#supplementary-data
