# Supplementary material for: Modulation of tooth regeneration through opposing responses to Wnt and BMP signals in teleosts
Source: Development. 2023 Dec 7;150(23):dev202168. doi: 10.1242/dev.202168 (PMC10730089; doi:10.1242/dev.202168)
Supplement: Supplementary file 1 [file develop-150-202168_review_history.pdf]

Modulation of tooth regeneration through opposing responses to Wnt and BMP signals in teleosts

Tyler A. Square, Emma J. Mackey, Shivani Sundaram, Naama C. Weksler, Zoe Z. Chen, Sujanya N. Narayanan and Craig T. Miller

DOI: 10.1242/dev.202168

Editor: Steve Wilson

Review timeline

Original submission:	13 July 2023
Editorial decision:	18 August 2023
First revision received:	9 October 2023
Accepted:	2 November 2023

Original submission

First decision letter

MS ID#: DEVELOP/2023/202168

MS TITLE: Modulation of tooth regeneration through opposing responses to Wnt and BMP signals in teleosts

AUTHORS: Tyler A Square, Emma J Mackey, Shivani A Sundaram, Naama C Weksler, Zoe Z Chen, Sujanya N Narayanan, and Craig T Miller

I have now received all the referees' reports on the above manuscript, and have reached a decision. The referees' comments are appended below, or you can access them online: please go to BenchPress and click on the 'Manuscripts with Decisions' queue in the Author Area.

As you will see, the referees express considerable interest in your work, but have some suggestions to improve your manuscript. If you are able to revise the manuscript along the lines suggested, I will be happy receive a revised version of the manuscript. Please also note that Development will normally permit only one round of major revision. If it would be helpful, you are welcome to contact us to discuss your revision in greater detail. Please send us a point-by-point response indicating your plans for addressing the referees' comments, and we will look over this and provide further guidance.

Please attend to all of the reviewers' comments and ensure that you clearly highlight all changes made in the revised manuscript. Please avoid using 'Tracked changes' in Word files as these are lost in PDF conversion. I should be grateful if you would also provide a point-by-point response detailing how you have dealt with the points raised by the reviewers in the 'Response to Reviewers' box. If you do not agree with any of their criticisms or suggestions please explain clearly why this is so.

Reviewer 1

Advance summary and potential significance to field

The manuscript is of interest to the evo-devo community, as it convincingly demonstrates that basic regulatory principles (here exemplified by an overall antagonism between Wnt- and Bmp signaling) are shared between epithelial organs in fish and mammals. The findings are carefully discussed,

© 2023. Published by The Company of Biologists under the terms of the Creative Commons Attribution License (https://creativecommons.org/licenses/by/4.0/).

this includes interesting ideas as to why the authors observed differences between zebrafish and stickleback tooth replacement following the overexpression studies. Overall, the manuscript contributes to a better understanding of the mechanisms active in teleost tooth replacement and discusses the results within a larger framework of how regenerative processes in epithelial appendages may have evolved by utilising the same genetic circuitry.

Comments for the author

This study examines the idea that the molecular signals controlling tooth and hair regeneration (examples of organ replacement from epithelial appendages) share genetic networks components. The authors tested if the opposing regulatory effects of Wnt and Bmp signaling in mammalian hair regeneration are controlling tooth replacing in threespine stickleback and zebrafish. They developed a two-stain pulse-chase protocol that allows them to tell which teeth developed during or before a time window, during which genetic manipulations took place in either species. The authors convincingly show that Wnt10a overexpression promotes the formation of new teeth in both species, while blocking Wnt signaling (through Dkk2 overexpression) blocks new tooth formation and fosters retention of old teeth. Bmp6 overexpression in stickleback stalled tooth formation and promoted shedding of existing teeth, therefore reducing total teeth number. Genes upregulated by Bmp6 overexpression were identified by RNAseq and confirmed the hypothesis that it causes inhibition of Wnt signaling, as expected from a hair regeneration model. The naïve dental epithelium was identified as a tissue where Wnt signaling is inhibited. Bmp pathway inhibition (through Grem2a overexpression or antagonist treatments) increased turnover of old teeth, while not affecting the rates of new tooth formation.
I have a few comments that the authors should address:
1. For a clearer understanding of the paper, especially for Figs. 1 and 4D,E, an overview of tooth formation should be provided that includes the stages and tissues in the process (ankylosis, bell- and eruption stage, tooth germ etc.).
2. Fig.8 and its legend do not match, there is no panel E.
3. The authors should also aim for a clearer understanding of the results by presenting a graphical model that summarizes their findings (as outlined in lines 358-370). In particular, the fact that Bmp inhibition does not promote new teeth, but decreased retained tooth number indicates that things are more complicated than the simple model where BMP- and WNT signaling antagonise each other in new and retained teeth. A visual summary would bring out the remaining questions more clearly.
4. A short note on whether scales, as another form of epithelial appendage are known to be controlled by Wnt- and Bmp-pathways in development or regeneration, would be appreciated.

<u>Reviewer 2</u>

Advance summary and potential significance to field

The manuscript by Square and coworkers takes a fresh look at tooth renewal in fish by contrasting the patterns to that of hair renewal. The use of both stickleback and zebrafish to perform overexpression assays (via heat-shock constructs) produce eloquent and clear results. The key aspect of the study is the separation of tooth renewal and tooth retention, thereby providing a comparative framework to hair renewal. Overal, this is an important study that raises the question whether there is a universal 'regeneration' program for all epithelia organs. This work should also stimulate and help researchers to identify organ specific aspects of regeneration.

Comments for the author

In the context of the thesis of the work, I consider the experimental evidence to be sufficient, and the discussion is well measured. To make the work more accessible, I have a couple of recommendations.
1) Part of the justification for the experimental work is the expression patterns of the studied genes (Fig. 1). Because the dynamic nature of tooth renewal in the fish, the Fig. 1 is not easy to interpret (for a non-fish researcher). My recommendation would be to include here a schematic figure showing bud and erupted teeth with the expression patterns of the four genes.

© 2023. Published by The Company of Biologists under the terms of the Creative Commons Attribution License (https://creativecommons.org/licenses/by/4.0/).

This would be especially useful for cross-organ comparisons. Incidentally, there is a new online tool available to examine expression patterns in the initiation of hair development: doi.org/10.1016/j.celrep.2023.112643
2) As the total number of teeth showed only marked increases in some of the zebrafish treatments, one wonders whether the lack of increase in stickleback could be in part due to limited space. That is, could tooth spacing or density become a limiting factor in the stickleback pharyngeal jaw that is packed with many teeth? And this relates to a concern I have about the interpretation of one aspect of the results: It seems that in treatments in which number of new teeth increased, the number of retained teeth always decreased (especially in the stickleback). How one can exclude the possibility that there is a density-dependent process going on that promotes the shedding of teeth as the function of increased generation of new teeth? Note that when you decrease the number of new teeth, higher or lower retention of teeth could be driven by many different processes. Regardless, I am not suggesting new experiments, or insist that the authors change their interpretations. This is just something to consider when finalizing the discussion.

Minor comments:
Not sure if the acronym SDE (successional dental epithelia) is needed as it is not mentioned many time and one forgets what it stands for.

Jukka Jernvall

Reviewer 3

Advance summary and potential significance to field

This paper looks at the molecular mechanisms controlling tooth replacement (number of teeth and rate of replacement) focusing on Bmps and Wnts.
The results shown are generally convincing and agree with results obtained in other models.
The finding that there is a molecular difference between overall number and rate is an important result.

Comments for the author

This paper aims to compare hair regeneration and tooth replacement, but this comes across as a bit forced. They only look at 4 genes, which have already been studied in the context of tooth development, so the need for the comparison is reduced. It is well known that epithelial Wnts stimulate tooth replacement, from studies in many vertebrates, while Wnt10a mutations in patients lead to hypodontia, so there is no need to base selection of this pathway on hair regeneration. Similarly, the authors have already published that an intronic enhancer of Bmp6 underlies tooth gain in sticklebacks (Cleves et al., 2018).
The similarities with the hair could be a discussion point but is really not the basis for selection of the genes analysed.

I, therefore, suggest a rewrite with the pathways being chosen based on the tooth literature, and a comparison to the hair added as a point of discussion, not the leading premise as currently in the abstract.

Figure 1 shows the expression pattern for the 4 genes selected.
Grem2a is very weak with lots of background. I suggest a repeat or use of a different method.
Wnt10a shows epithelial and mesenchymal expression from early stages of development, which is very different from the epithelial expression in the mouse during tooth development (Dassule and McMahon, 1998). This needs to be discussed.
Are the authors convinced this is Wnt10a, or a related Wnt?

A schematic of the successional dental epithelium would be useful to compare to the successional dental lamina of other species, as this is the area it would be predicted to be affected by the manipulations. The SDE is clear in figure 8, but its not clear where this region is in Figure 1 and how gene expression relates to it. Perhaps expression could be compared with the TCF/Lef construct in section for a direct comparison of expression in this region.

© 2023. Published by The Company of Biologists under the terms of the Creative Commons Attribution License (https://creativecommons.org/licenses/by/4.0/).

Figure 2 could probably be supplementary as it highlights the method used rather than any result. The technique though is very useful for differentiating tooth generations.

The overexpression experiments show significant changes that are adequately controlled for. However, counting teeth can be subjective. Did the authors do this blind with multiple observers to compare?

The use of two different teleosts is important for showing conservation of mechanisms across species. An image of the ZF teeth after Wnt10a overexpression and BMP6 would be useful to go with the graphs.

Figure 5 is really difficult to assess. I think better imaging is required to match teeth to the calcein dye.

The RNAseq data highlights an effect on Wnt signalling. This is then nicely confirmed using a TCF/Lef reporter.

I would divide the results into more headed sections for clarity

---

First revision

Author response to reviewers' comments

Dear reviewers and editors,

We thank you for the overall positive reviews and many helpful comments on our manuscript for *Development*. Below we provide detailed point-by-point responses to the reviewers' questions and suggestions, with reviewer comments in black and our responses underneath in bolded blue text.

Editor's comments to the authors:

As you will see, the referees express considerable interest in your work, but have some suggestions to improve your manuscript. If you are able to revise the manuscript along the lines suggested, I will be happy receive a revised version of the manuscript. Please also note that Development will normally permit only one round of major revision. If it would be helpful, you are welcome to contact us to discuss your revision in greater detail. Please send us a point-by-point response indicating your plans for addressing the referees' comments, and we will look over this and provide further guidance.

Reviewer 1

Advance Summary and Potential Significance to Field:
The manuscript is of interest to the evo-devo community, as it convincingly demonstrates that basic regulatory principles (here exemplified by an overall antagonism between Wnt- and Bmp signaling) are shared between epithelial organs in fish and mammals. The findings are carefully discussed, this includes interesting ideas as to why the authors observed differences between zebrafish and stickleback tooth replacement following the overexpression studies. Overall, the manuscript contributes to a better understanding of the mechanisms active in teleost tooth replacement and discusses the results within a larger framework of how regenerative processes in epithelial appendages may have evolved by utilising the same genetic circuitry.

Comments for the Author:

© 2023. Published by The Company of Biologists under the terms of the Creative Commons Attribution License (https://creativecommons.org/licenses/by/4.0/).

This study examines the idea that the molecular signals controlling tooth and hair regeneration (examples of organ replacement from epithelial appendages) share genetic networks components. The authors tested if the opposing regulatory effects of Wnt and Bmp signaling in mammalian hair regeneration are controlling tooth replacing in threespine stickleback and zebrafish. They developed a two-stain pulse-chase protocol that allows them to tell which teeth developed during or before a time window, during which genetic manipulations took place in either species. The authors convincingly show that Wnt10a overexpression promotes the formation of new teeth in both species, while blocking Wnt signaling (through Dkk2 overexpression) blocks new tooth formation and fosters retention of old teeth. Bmp6 overexpression in stickleback stalled tooth formation and promoted shedding of existing teeth, therefore reducing total teeth number. Genes upregulated by Bmp6 overexpression were identified by RNAseq and confirmed the hypothesis that it causes inhibition of Wnt signaling, as expected from a hair regeneration model. The naïve dental epithelium was identified as a tissue where Wnt signaling is inhibited. Bmp pathway inhibition (through Grem2a overexpression or antagonist treatments) increased turnover of old teeth, while not affecting the rates of new tooth formation.

I have a few comments that the authors should address:
1. For a clearer understanding of the paper, especially for Figs. 1 and 4D,E, an overview of tooth formation should be provided that includes the stages and tissues in the process (ankylosis, bell- and eruption stage, tooth germ, etc.).

We have added a new overview of tooth replacement (Fig. 10) which summarizes the major stages and tissues of tooth differentiation. We have also added a gene expression summary (Fig. 1O) which details the stages of tooth differentiation.

2. Fig.8 and its legend do not match, there is no panel E.

This has been corrected, we thank the reviewer for finding the error!

3. The authors should also aim for a clearer understanding of the results by presenting a graphical model that summarizes their findings (as outlined in lines 358-370). In particular, the fact that Bmp inhibition does not promote new teeth, but decreased retained tooth number indicates that things are more complicated than the simple model where BMP- and WNT signaling antagonise each other in new and retained teeth. A visual summary would bring out the remaining questions more clearly.

We have added a graphical model (Fig. 10) summarizing the effects of each ligand on different major tooth replacement events.

4. A short note on whether scales, as another form of epithelial appendage, are known to be controlled by Wnt- and Bmp-pathways in development or regeneration, would be appreciated.

A short note about fish scales and feathers was added to the introduction at line 73: "Scale regeneration in zebrafish has also been shown to be inhibited by *dkk1* overexpression (Aman et al., 2018), and feather regeneration in chickens has been shown to be inhibited by *Dkk2* overexpression (Chu et al., 2014), suggesting that Wnt signaling is required for the regeneration of a wide variety of epithelial organs."

Reviewer 2

<u>Advance Summary and Potential Significance to Field:</u>
The manuscript by Square and coworkers takes a fresh look at tooth renewal in fish by contrasting the patterns to that of hair renewal. The use of both stickleback and zebrafish to perform overexpression assays (via heat-shock constructs) produce eloquent and clear results. The key aspect of the study is the separation of tooth renewal and tooth retention, thereby providing a

© 2023. Published by The Company of Biologists under the terms of the Creative Commons Attribution License (https://creativecommons.org/licenses/by/4.0/).

comparative framework to hair renewal. Overal, this is an important study that raises the question whether there is a universal 'regeneration' program for all epithelia organs. This work should also stimulate and help researchers to identify organ specific aspects of regeneration.

Comments for the Author:
In the context of the thesis of the work, I consider the experimental evidence to be sufficient, and the discussion is well measured. To make the work more accessible, I have a couple of recommendations.

1) Part of the justification for the experimental work is the expression patterns of the studied genes (Fig. 1). Because the dynamic nature of tooth renewal in the fish, the Fig. 1 is not easy to interpret (for a non-fish researcher). My recommendation would be to include here a schematic figure showing bud and erupted teeth with the expression patterns of the four genes. This would be especially useful for cross-organ comparisons. Incidentally, there is a new online tool available to examine expression patterns in the initiation of hair development:
doi.org/10.1016/j.celrep.2023.112643

We have added a new diagram (Fig. 1M) summarizing the gene expression found at key tooth stages for the four genes studied here.

2) As the total number of teeth showed only marked increases in some of the zebrafish treatments, one wonders whether the lack of increase in stickleback could be in part due to limited space. That is, could tooth spacing or density become a limiting factor in the stickleback pharyngeal jaw that is packed with many teeth? And this relates to a concern I have about the interpretation of one aspect of the results: It seems that in treatments in which number of new teeth increased, the number of retained teeth always decreased (especially in the stickleback). How one can exclude the possibility that there is a density-dependent process going on that promotes the shedding of teeth as the function of increased generation of new teeth? Note that when you decrease the number of new teeth, higher or lower retention of teeth could be driven by many different processes. Regardless, I am not suggesting new experiments, or insist that the authors change their interpretations. This is just something to consider when finalizing the discussion.

We agree and appreciate this thoughtful suggestion. At line 466 in the Discussion we added "Alternatively, given that zebrafish were able to demonstrate an increase in total tooth number (via *wnt10a* OE), while sticklebacks did not demonstrate this capability, these different responses could instead suggest that some space or density constraints may exist in sticklebacks that do not in zebrafish."

Minor comments:
Not sure if the acronym SDE (successional dental epithelia) is needed as it is not mentioned many time and one forgets what it stands for.

We have removed the SDE acronym from the main text.

Jukka Jernvall

Thank you, Dr. Jernvall!

Reviewer 3

Advance Summary and Potential Significance to Field:
This paper looks at the molecular mechanisms controlling tooth replacement (number of teeth and rate of replacement) focusing on Bmps and Wnts. The results shown are generally convincing

© 2023. Published by The Company of Biologists under the terms of the Creative Commons Attribution License (https://creativecommons.org/licenses/by/4.0/).

and agree with results obtained in other models. The finding that there is a molecular difference between overall number and rate is an important result.

Comments for the Author:
This paper aims to compare hair regeneration and tooth replacement, but this comes across as a bit forced. They only look at 4 genes, which have already been studied in the context of tooth development, so the need for the comparison is reduced. It is well known that epithelial Wnts stimulate tooth replacement, from studies in many vertebrates, while Wnt10a mutations in patients lead to hypodontia, so there is no need to base selection of this pathway on hair regeneration. Similarly, the authors have already published that an intronic enhancer of Bmp6 underlies tooth gain in sticklebacks (Cleves et al., 2018). The similarities with the hair could be a discussion point but is really not the basis for selection of the genes analysed. I, therefore, suggest a rewrite with the pathways being chosen based on the tooth literature, and a comparison to the hair added as a point of discussion, not the leading premise as currently in the abstract.

> **To better cite the previous literature on tooth functional studies, the following text now appears in the introduction before outlining the "hair motivation," starting at line 66: "Functional studies on tooth and other epithelial appendage development previously implicated a positive role for Wnt/ß-catenin signaling in these processes. Stimulation of downstream Wnt/ß-catenin signaling via a constitutively active ß-catenin (*Ctnnb*) has been shown to induce mouse molar regeneration, a process which normally does not occur in this species, supporting a crucial role for Wnt signaling in promoting whole tooth organ regeneration (Järvinen et al., 2006; Popa et al., 2019). Recombinant human WNT3A was shown to cause increased cell division in the dental lamina of alligator jaw explants (Wu et al., 2013). Scale regeneration in zebrafish has also been shown to be inhibited by *dkk1* overexpression (Aman et al., 2018), and feather regeneration in chickens has been shown to be inhibited by *Dkk2* overexpression (Chu et al., 2014), suggesting that Wnt signaling is required for the regeneration of a wide variety of epithelial organs."**
>
> **We respectfully disagree with Reviewer 3 on the leading premise of this study and the true basis for the selection of the genes studied here. "Testing the genetic conservation of tooth and hair replacement" was the title of the first author's NIH Fellowship from the National Institute for Dental and Craniofacial research (https://reporter.nih.gov/search/xXDPlNoy7Ue2Qpi55yU0cw/project- details/9886077), and other work related to this hypothesis is currently funded by the last author's R01 from the same Institute. Making the comparison between hair and teeth was our primary objective in designing these experiments and selecting genes. We agree with the reviewer that the Wnt and BMP pathways have generally been shown to affect tooth development, but here our question is about the potential sufficiency of specific ligands (per the last sentence of the first introduction paragraph, line 46: "However, little is known about which specific ligands can regulate the process of whole tooth replacement."). The WNT3A alligator explant experiment is one of the few where the effects of a particular Wnt ligand were tested using gain-of-function - this study is now cited. We feel that our explained framing of this work in the abstract of the paper is both true to the history of this project and is logically sound.**

Figure 1 shows the expression pattern for the 4 genes selected. Grem2a is very weak with lots of background. I suggest a repeat or use of a different method.

> **We have replaced former panels in Fig. 1L and M with new panels L, M, and N with better signal-to-noise ratio of *Grem2a in situ* on sectioned stickleback pharyngeal tissue.**

© 2023. Published by The Company of Biologists under the terms of the Creative Commons Attribution License (https://creativecommons.org/licenses/by/4.0/).

Wnt10a shows epithelial and mesenchymal expression from early stages of development, which is very different from the epithelial expression in the mouse during tooth development (Dassule and McMahon, 1998). This needs to be discussed. Are the authors convinced this is Wnt10a, or a related Wnt?

To address this timing difference of *Wnt10a* expression in mouse and fish teeth, at line 349 we added the text: "Notably, unlike the stickleback mesenchymal domain, the mesenchymal domain of *Wnt10a* in the mouse incisor appears stronger at later stages of tooth development than earlier stages (Dassule and McMahon, 1998; Suomalainen and Thesleff, 2010), suggesting a temporal shift in the expression of this ligand between mice and fish."

We are confident that the *Wnt10a* genes reported here are the stickleback and zebrafish orthologs of the mammalian *Wnt10a* gene based on synteny (e.g. *Wnt10a* is flanked by the *Wnt6b* and *cdk5r2* genes in sticklebacks, zebrafish, mice, and humans). The sequences of these fish *Wnt10a* genes are also the reciprocal best BLAST match to mammalian Wnt10a, further supporting their orthology. Link to synteny browser: https://www.genomicus.bio.ens.psl.eu/genomicus-100.01/cgi-bin/phyloview.pl?&root=7070757&id=7071238

A schematic of the successional dental epithelium would be useful to compare to the successional dental lamina of other species, as this is the area it would be predicted to be affected by the manipulations. The SDE is clear in figure 8, but its not clear where this region is in Figure 1 and how gene expression relates to it. Perhaps expression could be compared with the TCF/Lef construct in section for a direct comparison of expression in this region.

We have added a new Fig. 10 that diagrams the location of the successional dental epithelium. We have also added an additional point of clarification to the Figure 1 legend directing the reader to new Fig. 10 of this paper and a similar diagram in Square et al. 2021 where we describe this region in detail, at line 721: "Descriptions of the location of the successional dental epithelium can be found in Fig. 10 in this paper and Fig. 6 in Square *et al*., 2021."

Figure 2 could probably be supplementary as it highlights the method used rather than any result. The technique though is very useful for differentiating tooth generations.

We moved panels A and B of Fig. 2 to the supplementary materials (now in Fig. S2) but left the pulse-chase images (formerly Fig. 1C and D) as mentioned by the reviewer as illustrative. We feel it is important for the reader's understanding of the method to see a close-up of these pulse- chase labeled tooth fields, given how central this method is to most of the quantifications of tooth replacement in this study.

The overexpression experiments show significant changes that are adequately controlled for. However, counting teeth can be subjective. Did the authors do this blind with multiple observers to compare?

We have added additional clarification at line 163 that all skeletal preparations were scored blindly: "We thus coupled OE treatments of Wnt10a, Dkk2, Bmp6, or Grem2a with a two-color pulse-chase bone staining protocol (Figs 2 and S1) scored blindly, allowing us to classify every tooth in each fish as either "new" or "retained" with respect to the OE treatment interval (see Methods)." A single observer (the first author) scored all the samples in this paper to reduce the subjectiveness of scoring between experiments. Because the calcein dye fades once the experiment is complete, we invoked strict parameters regarding when each experiment was scored

© 2023. Published by The Company of Biologists under the terms of the Creative Commons Attribution License (https://creativecommons.org/licenses/by/4.0/).

upon completion (from Methods line 619: "Skeletal preparations from a given experiment were thus always scored within two days of the end of each experiment, and always within a single 24 hour window"). Given that it sometimes takes more than 12 total hours to score 20+ sticklebacks, and that zebrafish samples are destroyed as they are scored (see next response), it was not feasible to have multiple researchers score entire experiments.

The use of two different teleosts is important for showing conservation of mechanisms across species. An image of the ZF teeth after Wnt10a overexpression and BMP6 would be useful to go with the graphs. Figure 5 is really difficult to assess. I think better imaging is required to match teeth to the calcein dye.

We added more annotation (white circles) to Fig. 5D to better highlight tooth germ positions. We also added images of zebrafish *wnt10a* overexpression as new Fig. S10 along with a more thorough explanation of our dissection protocol in the methods at line 632: "In order to accurately score zebrafish teeth, it was necessary to remove the epithelial layer from the underlying bony tissues. Tooth germs would typically stay embedded within this epithelial layer (Fig. S10). Thereafter, teeth in the dorsal and middle rows were often broken off to be viewed in isolation."

The RNAseq data highlights an effect on Wnt signalling. This is then nicely confirmed using a TCF/Lef reporter. I would divide the results into more headed sections for clarity

We added and updated subheadings of the Results between lines 198-291. The subheadings now read: "Wnt pathway stimulation by overexpression of *Wnt10a*" (line 175), "Wnt pathway inhibition by overexpression of *Dkk2*" (line 198), "BMP pathway stimulation by overexpression of *Bmp6*" (line 214), "*Bmp6* overexpression causes Wnt signaling inhibition in successional dental epithelia" (line 249), "BMP pathway inhibition by overexpression of *Grem2a* and LDN-192189" (line 291).

Together we think these revisions have substantially improved our manuscript, which we now hope is suitable for publication in *Development*. Please let us know if you have any questions.

Sincerely,

Dr. Tyler Square
Dr. Craig Miller

Molecular & Cell Biology Department University of California, Berkeley

---

Second decision letter

MS ID#: DEVELOP/2023/202168

MS TITLE: Modulation of tooth regeneration through opposing responses to Wnt and BMP signals in teleosts

AUTHORS: Tyler A Square, Emma J Mackey, Shivani A Sundaram, Naama C Weksler, Zoe Z Chen, Sujanya N Narayanan, and Craig T Miller
ARTICLE TYPE: Research Article

© 2023. Published by The Company of Biologists under the terms of the Creative Commons Attribution License (https://creativecommons.org/licenses/by/4.0/).

I am happy to tell you that your manuscript has been accepted for publication in Development, pending our standard ethics checks.

Reviewer 1

Advance summary and potential significance to field

As stated previously, the manuscript contributes to a better understanding of the mechanisms active in teleost tooth replacement and discusses the results within a larger framework of how regenerative processes in epithelial appendages may have evolved by utilising the same genetic circuitry.

Comments for the author

The authors have addressed all of my comments and improved the manuscript. I have no further issues that need to be addressed before possible publication.

Reviewer 2

Advance summary and potential significance to field

I find the revision improved and ready to publish.

Comments for the author

The authors have addressed all my comments. I find the new schematic figures 1 and 10, and figures in general, very clear and the paper is now much easier to follow. The discussion is well measured.

Reviewer 3

Advance summary and potential significance to field

The revised manuscript is much clearer and the additional schematics really help.
I am happy that the authors have addressed my comments.

Comments for the author

None

© 2023. Published by The Company of Biologists under the terms of the Creative Commons Attribution License (https://creativecommons.org/licenses/by/4.0/).
